# How Do Soil Bacterial Diversity and Community Composition Respond under Recommended and Conventional Nitrogen Fertilization Regimes?

**DOI:** 10.3390/microorganisms8081193

**Published:** 2020-08-05

**Authors:** Sami Ullah, Ping He, Chao Ai, Shicheng Zhao, Wencheng Ding, Dali Song, Jiajia Zhang, Shaohui Huang, Tanveer Abbas, Wei Zhou

**Affiliations:** Ministry of Agriculture Key Laboratory of Plant Nutrition and Fertilizer, Institute of Agricultural Resources and Regional Planning, Chinese Academy of Agricultural Sciences (CAAS), Beijing 100081, China; samisheen14@yahoo.com (S.U.); aichao@caas.cn (C.A.); zhaoshicheng@caas.cn (S.Z.); wcding@126.com (W.D.); songdali@caas.cn (D.S.); zhangjiajia_91@163.com (J.Z.); shaohui1988@sina.com (S.H.); abbastanveer272@gmail.com (T.A.); zhouwei02@caas.cn (W.Z.)

**Keywords:** Nitrogen fertilization, bacterial diversity, bacterial community structure, soil properties

## Abstract

Shifts in soil bacterial diversity and community composition are suggested to be induced by elevated input of nitrogen (N) fertilization with implications for soil quality, and consequently production. In this study, we evaluated the impacts of recommended fertilization (RF) and conventional fertilization (CF) on soil chemical properties, crop yield, bacterial diversity, and community composition from two long-term experiments conducted in fluvo-aquic soil and black soil of China. Each site comprised of four treatments, i.e., RF N−, RF N+, CF N−, CF N+. No N fertilization was indicated by N− and N fertilization was indicated by N+. Across both sites, N fertilization significantly increased crop yield compared with no N fertilization and RF successfully enhanced crop yield over CF. Interestingly, the RF maintained bacterial diversity, while CF depressed bacterial diversity in the two soils. Microbial taxa performing important ecological roles such as order Rhodospirillales and Bacillales were significantly enhanced in the RF approach, while *Rhizobiales* declined under CF. Furthermore, the results of partial least square path modeling revealed that soil available phosphorus (AP) negatively affected bacterial diversity while it positively affected bacterial community structure in fluvo-aquic soils. In contrast, soil pH was positively linked with both bacterial diversity and community structure in black soil. Overall, our study demonstrated that RF is an environmentally friendly approach which not only maintained above ground plant productivity, but also preserved belowground microbial populations and important soil variables regulating bacterial communities varied in different soil types.

## 1. Introduction

Mineral fertilizers, especially nitrogen (N), constitute a large contributor to global reactive N input, which was 86 Tg N in 1995 and was predicted to hit 135 Tg N by 2050 [1,2]. Understanding how elevated N enrichment will affect terrestrial ecosystems is becoming more importance [3]. N is the limiting nutrient for the majority of crops as well as for terrestrial ecosystems [4], and chronic N input can exert strong impacts on plant community composition and above ground primary production [4]. However, below-ground, microbial communities are also impacted by elevated addition of nutrients [5], but they have been less well understood. Soil microbial communities are fundamental components of terrestrial ecosystems and are directly or indirectly engaged in biogeochemical processes, particularly N transformation and cycling, decomposition of organic matter, and other ecosystem services and functions [6]. It is well documented that a change in microbial community structure in response to elevated N addition influences global carbon (C) and N cycling [6]. N addition also results in reduced microbial activity due to reduced soil respiration and/or an increase in C sequestration [7]. However, it remains uncertain if shifts in microbial communities are directly associated with N enrichment or indirectly due to changes in soil properties and plant communities [8]. Microbial diversity is very imperative to ecological functions and sustaining soil productivity in arable lands [9]. Recent work has shown that chronic N enrichment results in declined microbial diversity [10]. However, the subsidy-stress hypothesis states that higher levels of N addition result in reduced diversity, while moderate levels of amendment may promote microbial diversity [11,12].

It is well established that changes in soil properties followed by N fertilization such as, soil acidification and nutrient availability can cause ambient shifts in microbial diversity and community composition [13]. However, studies showed that important soil properties causing shifts in bacterial community composition and diversity varied for example, in a long-term inorganic fertilizer experiment showed that, soil acidification (soil pH) directly impacted bacterial community composition but not soil organic carbon (SOC) [14]. On the other hand, a study confirmed that N addition directly impacted bacterial diversity by increasing soil N availability while indirectly affected bacterial community composition through soil acidification [15]. Other studies showed that, soil pH is considered as main predictor in shaping bacterial community composition and diversity [10,13]. In addition, it was claimed that soil available phosphorus (AP) significantly influenced bacterial richness and community composition but not bacterial diversity [16]. Alternatively, findings suggested that AP did not significantly affect bacterial richness and diversity, but N availability significantly affected bacterial richness and diversity [17].

With a goal of higher yields, Chinese farmers tend to apply high quantities of fertilizers, which not only degrade soil quality but also induced shifts in soil microbial communities [18]. To preserve the ecological integrity of cropland and to address the challenge of rising food demands, sustainable nutrient management strategies must be developed. Thus, the objectives of the present study were to (i) assess the impact of recommended fertilization (RF) and conventional fertilization (CF) on maize yield, soil bacterial diversity, and community structure, (ii) to identify which bacterial taxa will be significantly affected by recommended and conventional fertilization, and (iii) identify important soil variables influencing bacterial diversity and community structure in fluvo-aquic and black soils. We hypothesized that (i) unlike reduced bacterial diversity under elevated N inputs, recommended fertilization would preserve bacterial diversity, because higher levels of N addition result in reduced diversity, while moderate levels of amendment may promote microbial diversity [11,12], (ii) recommended fertilization would improve beneficial bacterial taxa performing important ecological functions, and (iii) key soil properties affecting bacterial diversity and community structure will be different in black soil and fluvo-aquic soil respectively because, site-specific dynamics can strongly shape bacterial diversity [19] and community structure [20].

## 2. Materials and Methods

### 2.1. Experimental Sites

The long-term field trial was established in 2009 at the Dahe Experimental Station located in Shijiazhuang City, Hebei Province (38°07ʹ N and 114°29ʹ E) north central China. That region has fluvo-aquic soil (Calcaric Cambisol, FAO). The study area has typical warm temperate and sub-humid continental monsoon climate. The average annual temperature is 14.3 °C and precipitation is 400 mm. Another field trial was established in 2009 in Liufangzi County, Gongzhuling City, Jilin Province (43°34.86ʹ N and 124°53.92ʹ E) northeastern China. This study area has black soils and is called as (Haplic Phaeozems, FAO) and as a Mollisol in the USDA classification. This site has a temperate and semi-humid continental monsoon climate. The average annual temperature is 4–5 °C. The mean annual precipitation is 500–900 mm. This study area has black soils and is called as (Haplic Phaeozems, FAO).

### 2.2. Experimental Design

In both study locations, the experiment was conducted in randomized complete block design with four replicates, and each subplot was 45 m^2^ (5 × 9 m) and 40 m^2^ (10 × 4 m) in fluvo-aquic soil and black soil site, respectively. There were four treatments in fluvo-aquic soil: RF N0, RF N182, CF N0, CF N225 kg ha^−1^. Similarly, four treatments were conducted in black soils, namely RF N0, RF N180, CF N0, and CF N251 kg ha^−1^ (Table 1). In total, there were 32 experimental plots (4 treatments × 4 rep × 2 sites). Furthermore, in both sites in the RF treatments one-quarter of the N and all the P and K was provided in a basal application with the remaining N was applied at critical growth stages of the crop such as heading and tasseling. In contrast, the CF treatments represented the average fertilization rate applied by farmers in north-central (fluvo-aquic soil) and northeast (black soil) China, with all of the N, P, and K provided in a basal application. The source of N in this study was urea. No organic fertilizer source was used. The remaining agronomic practices such as tillage and pesticide application remained same for recommended fertilization and conventional fertilization treatments. The cropping rotation system was winter wheat-summer maize in north-central China and summer maize mono cropping system was practiced in northeast China.

### 2.3. Sampling, Determination of Soil Bio-Chemical Properties and Maize Yield

Soil samples were collected in September 2016 from both locations at maize harvest. Generally, five soil cores (2 cm in diameter) were collected from each experimental plot to a depth of 0–20 cm and pooled to create a composite sample. One part of the subsample was held at −80 °C for further molecular analysis. The nitrate-nitrogen (NO_3_^−^ N) and ammonium-nitrogen (NH_4_^+^ N) were extracted from a 12 g fresh soil sample using a 1:10 ratio of soil to 0.01 mol L^−1^ CaCl_2_ solution and then analyzed using continuous flow analysis (Foss FlAstar 5000). The determination of soil water content was conducted by oven drying soil at 105 °C. Soil total nitrogen (TN) and soil organic carbon (SOC) was analyzed by Kjeldahl digestion [21] and dichromate oxidation respectively [22]. The Olsen method was used to detect available phosphorus (AP) [23]. The soil pH was measured with a combination electrode (PE-10, Sartorius, Germany) using a soil:water ratio of 1:2.5. The C and N in microbial biomass was carried out with fumigation extraction method with 0.5 M K_2_SO_4_ and determined by a total organic C/N analyzer (Multi N/C3100/HT1300, Analytik Jena AG, Jena, Germany) [24]. In late September, when the maize matured, a sampling area was randomly selected from each experimental plot to evaluate grain yield. We selected two middle rows from each experimental plot of maize, and ten maize plants were collected to determine their moisture content. This was converted to a standard moisture content of 15.5% to measure the final grain yield.

### 2.4. Bioinformatics Analyses

#### 2.4.1. DNA Extraction and PCR Amplification

Genomic DNA was extracted from 0.5 g soil samples using the E.Z.N.A. ^®^ Bacterial DNA Kit (Omega Biotek, Norcross, GA, USA) according to the manufacturer’s instructions. DNA quality was tested by 1% agarose gel electrophoresis and spectrophotometry (optical density at 260 nm/280 nm ratio). 16S rRNA gene V3-V4 hypervariable regions were sequenced with Illumina MiSeq PE300 sequencing (Illumina, Inc., San Diego, CA, USA). The pair of universal primers 338F (5′ ACTCCTACGGGAGGCAGCAG-3) and 806R (5′ GACTACHVGGGTWTCTAAT-3′) was used to amplify V3-V4 regions of 16S rRNA. Conditions for the PCR amplification were as follows: 95 °C for 5 min, and 25 cycles at 95 °C for 30 s, 55 °C for 30 s, and at 72 °C for 30 s with a final extension of 72 °C for 10 min. PCR was conducted in triplicate in a 25 μL mixture containing 2.5 μL of 10 × Pyrobest buffer, 2 μL of 2.5 mM dNTPs, 1 μL of each primer (10 μM), 0.4 U of Pyrobest DNA polymerase (TaKaRa), and 15 ng of template DNA. Amplicon mixtures were transfered to the MiSeq Genome Sequencer (Illumina, San Diego, CA, USA).

#### 2.4.2. Illumina MiSeq Sequencing

Amplicons were extracted from 2% agarose gels, purified using the AxyPrep DNA Gel Extraction Kit (Axygen Biosciences, Union City, CA, USA) according to the manufacturer’s instructions and quantified using QuantiFluor™ -ST (Promega, Madison, WI, USA). We pooled purified amplicons in equimolar and paired-end sequences (2 × 300) on an Illumina MiSeq platform according to the standard protocols. High-quality sequence extraction was conducted with the Quantitative Insights into Microbial Ecology (QIIME) (v1.2.1) package. The raw sequences were selected, and the sequences with low-quality were eliminated (i) if raw reads were shorter than 110 nucleotides; (ii) if 300 bp reads were truncated at any site receiving an average quality score <20 over a 50 bp sliding window, discarding the truncated reads that were shorter than 50 bp; or (iii) if exact barcode matching or a two nucleotide mismatch in primer matching were obtained and reads contained ambiguous characters; only sequences that overlap more than 10 bp were assembled according to their overlap sequence. Reads that could not be assembled were discarded. The unique sequence set was classified into operational taxonomic units (OTUs) under the threshold of 97% identity using UCLUST. Chimeric sequences were identified and removed using USEARCH (version 8.0.1623). The taxonomy of each 16S rRNA gene sequence was analyzed by UCLUST against the Silva119 16S rRNA database using a confidence threshold of 90%.

### 2.5. Statistical Analyses

For each variable measured (e.g., soil chemical properties or bacterial alpha diversity indices) the data were analyzed by one-way analysis of variance (ANOVA) using Fisher’s least significant difference (LSD) at (*p* ≤ 0.05) to compare the treatment means. Two-way ANOVA was used to evaluate the effect of treatments and soil types on soil biochemical traits, maize yield, and alpha diversity index. To identify taxa which were significantly affected by RF and CF, we carried out the linear discriminant analysis (LDA) effect size (LEfSe) algorithm [25], biomarkers examined in this study are consistent with the following standards: (i) minimum LDA score (log10 value) for discriminative features are ≥2.5 and (ii) alpha value for the factorial Kruskal-Wallis test between groups ≤0.05. Aggregated boosted trees (ABT) analysis, a statistical tool that aims to obtain both explanation and accurate predictions, was performed using the ‘gbmplus’ package (with 500 trees for the boosting, a 0.02-fold shrinkage rate and three-way interactions) with the R software version 2.7.1 [26]. We further explored the complex relationship among soil properties, maize yield, bacterial diversity, and community structure using partial least squares path modeling (PLSPM). A statistical tool was used to show the cause and affect relationships among the observed variables and latent variables [27]. Path coefficients (direct effect) indicated the direction and strength of the linear relationship between variables, whereas indirect effects indicated multiplied path coefficients between a predictor and a response variable, adding the product of all possible paths except the direct effect [28]. The estimates of the coefficients of determination (R^2^) and the estimate of path coefficient in our model were validated using “plspm” package (1000 bootstraps) in R software version 3.4.4. Nonmetric multidimensional scaling (NMDS) based on Bray–Curtis dissimilarities was employed to identify shifts in soil bacterial community structures. 

## 3. Results

### 3.1. Soil Chemical, Biological Properties, and Crop Yield

**Figure 1:**
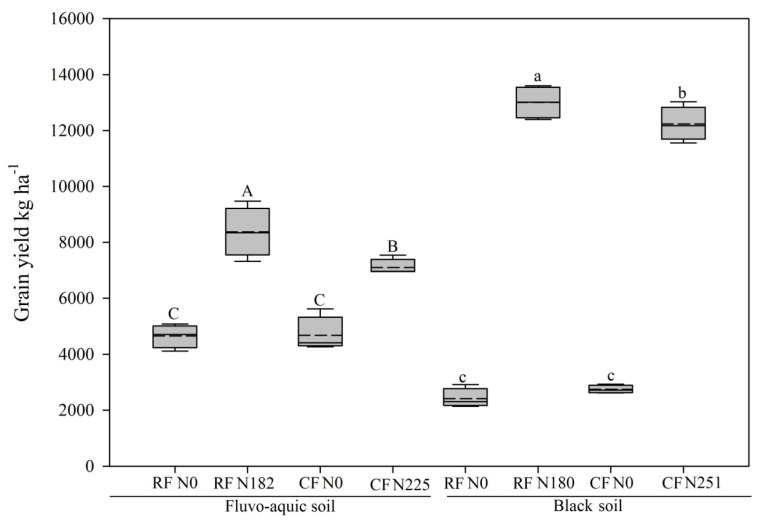
Maize yield in four fertilization treatments and two soil types. Box plots show median (horizontal line), mean (dotted line), 25th and 75th percentiles (box), and 5th and 95th percentiles (whisker caps). Capital letters are used to indicate significance for fluvo-aquic soil and small letters are used for black soil.

In fluvo-aquic soil, N fertilization (*p* ≤ 0.05) significantly reduced the soil pH and lowest pH was found in CF N225 treatment (Table 2). Soil TN significantly increased as a result of N fertilization (*p* ≤ 0.05), and highest TN was recorded in RF N182 treatment. Nitrate (NO_3_^−^ N) levels significantly increased as a result of the N addition (*p* ≤ 0.01), with the highest values observed in CF N225. By contrast, N fertilization (*p* ≤ 0.05) significantly decreased AP, and the lowest AP was found in RF N182. N fertilization did not significantly influence NH_4_^+^ N, SOC, C:N ratio, MBC, and MBN stocks. In black soil, soil pH significantly declined as a result of the N addition (*p* ≤ 0.01; Table 2), and the lowest pH was found in CF N251 treatment. Conversely, TN significantly increased due to the effects of N fertilization (*p* ≤ 0.001) and highest value was found in the CF N251. Furthermore, NO_3_^−^ N levels increased as a result of the N fertilization (*p* ≤ 0.01), and highest level was found in the RF N180 treatment. Ammonium levels increased as a result of N fertilization (*p* ≤ 0.01). MBC was declined as a result of N fertilization (*p* ≤ 0.05) and lowest MBC was recorded in the CF N251 treatment. Both the MBN and C:N ratio declined as a result of N fertilization (*p* ≤ 0.05; *p* ≤ 0.001), with the lowest values observed in CF N251 treatment. N fertilization did not significantly impacted SOC stocks. The maize yield was evaluated (Figure 1). The results of the box plot showed that, in comparison with the CF treatments, the RF treatments receiving N successfully enhanced the maize yield in the two soils. In fluvo-aquic soil, RF N182 treatment produced a 13.15% greater yield than did CF N225 treatment. In black soil, RF N180 treatment produced a 6.31% greater yield than did CF N251treatment. The effect of N fertilization, soil type, and their interaction with biochemical properties and crop yield are presented in Table 3.

### 3.2. Bacterial Alpha Diversity under Long-Term Fertilization

**Figure 2:**
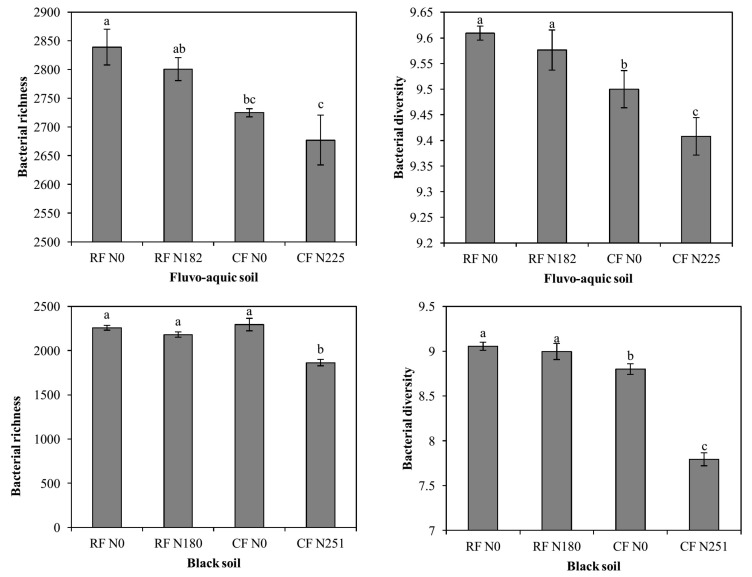
Comparison of estimated alpha diversity indices of 16s rRNA gene libraries for clustering at 97% similarity, obtained from high throughput sequencing analysis. Data are the means, n = 4, error bars represents standard error. Bars with the same letter are not significantly different at *p* ≤ 0.05.

In the present study, the Shannon index [29] and Chao1 [30] were used to investigate bacterial diversity and richness, respectively. In fluvo-aquic soil, the results showed that the N fertilization significantly altered bacterial richness (Chao1) (Figure 2). Bacterial taxonomic diversity (Shannon index) was significantly altered due to the N fertilization (*p* ≤ 0.05). In black soil, the N Fertilization altered (*p* ≤ 0.01) bacterial richness and taxonomic diversity. Interestingly, at both locations, in the RF treatments, bacterial diversity was maintained, but in CF treatments, there was a gradual decline in bacterial diversity and richness that paralleled N addition, and lowest values were found in the CF N225 and CF N251 treatments respectively. The effect of N fertilization, soil type, and their interaction with the bacterial alpha diversity index is shown in the Table 3.

### 3.3. Bacterial Community Composition under Long-Term Fertilization

**Figure 3:**
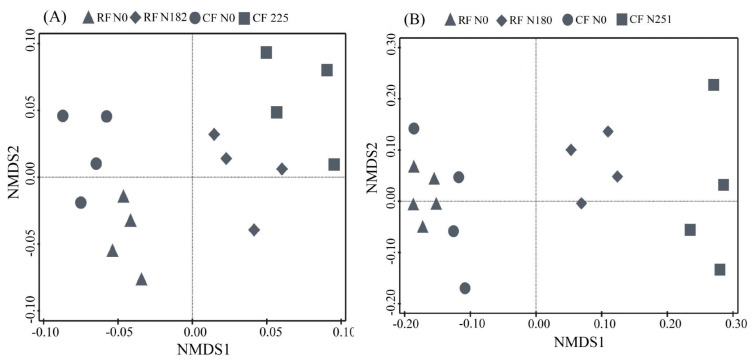
Non-metric multidimensional scaling (NMDS) of bacterial communities under long-term fertilization in fluvo-aquic soil (**A**), and black soil (**B**), based on Bray-Curtis dissimilarities.

**Figure 4:**
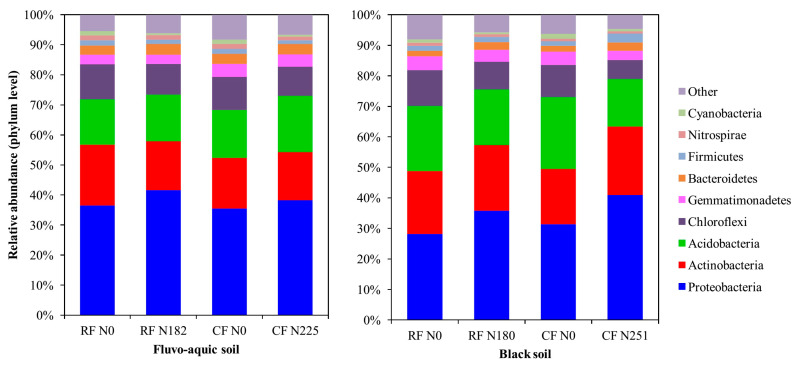
The relative abundances of dominant bacterial groups (phylum level) in fluvo-aquic soil and black soil, based on the proportional frequencies of 16S rRNA sequences.

To determine whether the management system and N fertilization led to shifts in soil bacterial communities, we profiled the structural changes in bacterial communities using NMDS based on Bray-Curtis dissimilarities. It was evident from the NMDS ordinations that there was clear separation among bacterial communities associated with recommended and conventional N fertilizer rates in the two soils (Figure 3). Bacterial taxonomic compositions were evaluated. Proteobacteria, Actinobacteria, Acidobacteria, and Chloroflexi were the most dominant phyla in both locations (Figure 4). In fluvo-aquic soil, the results showed that the abundance of phylum Actinobacteria significantly increased, while Nitrospirae decreased as a result of N addition. In black soil, results revealed that, the abundance of Proteobacteria and Bacteroidetes significantly increased as a result of N fertilization, while N fertilization significantly decreased the abundance of Chloroflexi.

**Figure 5:**
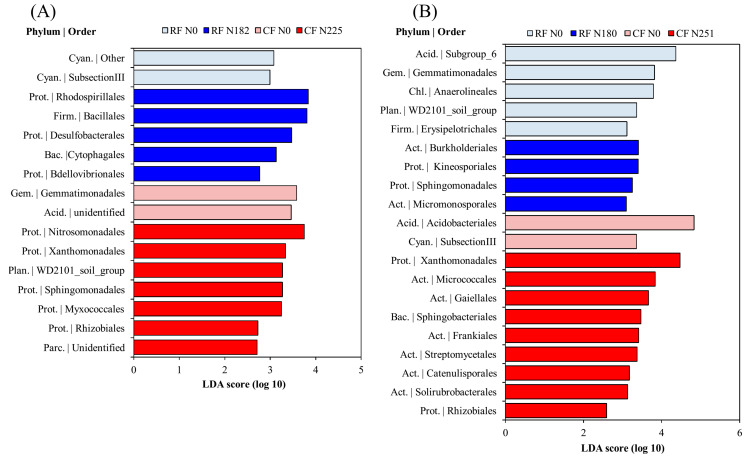
Bacterial key phylotypes (at the order level) significantly altered under RF and CF fertilization identified using linear discriminant analysis (LDA) effect size (LEfSe) in fluvo-aquic soil (**A**), black soil (**B**). Abbreviations: Acid. = Acidobacteria; Act. = Actinobacteria; Bac. = Bacteroidetes; Cyan. = Cyanobacteria; Chl. = Chloroflexi; Firm. = Firmicutes; Gem. = Gemmatimonadetes; Parc. = Parcubacteria; Plan. = Planctomycetes; Prot. = Proteobacteria.

We further identified high-dimensional biomarkers at the order level using LEfSe, a robust algorithm that recognizes features among biological groups that are significantly different (Segata et al., 2011), and explored which taxa were significantly altered under RF and CF treatments. In fluvo-aquic soil, Rhodospirillales (LDA score = 3.83) and Bacillales (LDA score = 3.80) were significantly altered taxa in RF N182 treatment (Figure 5A). Conversely, Nitrosomonadales (LDA score = 3.74) and Xanthomonadales (LDA score = 3.33) were significantly altered in CF N225. In black soil, order subgroup_6 (LDA score = 4.36) belonging to phylum Acidoacteria and Acidobacteriales (LDA score = 4.83) were sensitive to N fertilization, and its abundance was highest in RF N0 and CF N0 treatments respectively (Figure 5B). Interestingly, the predominant order Xanthomonadales (LDA score = 4.47) was significantly altered in CF N251 treatment. Across both soils, elevated N inputs (CF N225 and CF N251 treatments) significantly reduced order Rhizobiales.

### 3.4. Bacterial Diversity and Community Composition in Relation to Soil Properties

**Figure 6:**
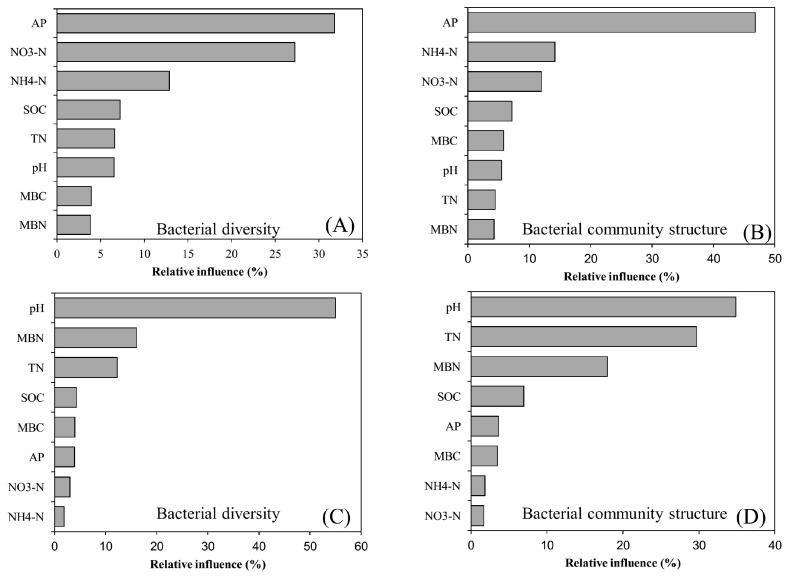
Relative influence of the driving factors for bacterial diversity and community structure as determined by aggregated boosting tree (ABT) analysis, in fluvo-aquic soil (**A**,**B**), and black soil (**C**,**D**). TN: total nitrogen, SOC: soil organic carbon, AP: available phosphorus, NO_3_^−^ N: nitrate-N, NH_4_^+^ N: ammonium-N, MBC: microbial biomass carbon, MBN: microbial biomass nitrogen.

**Figure 7:**
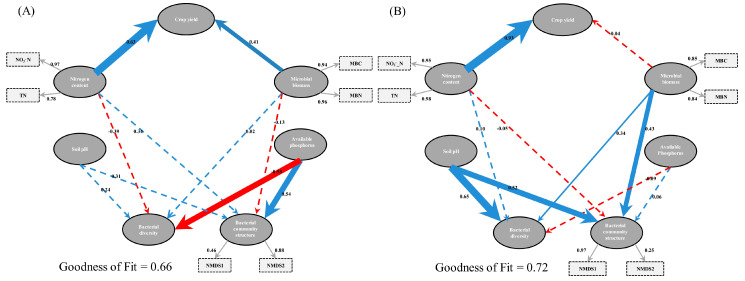
Directed graph of the partial least squares path model (PLS-PM). Each oval shape represents an observed variable (i.e., measured) and box represents latent variable (i.e., constructs). The loading for nonmetric multidimensional scaling (NMDS) scores of bacterial communities, microbial biomass carbon (MBC), microbial biomass nitrogen (MBN), total nitrogen (TN) and NO_3_^−^ N that create the latent variables are shown in the dashed rectangle. Path coefficients are calculated after 1000 bootstraps and reflected in the solid width of the arrow, with blue and red indicating positive and negative effects, respectively. Dashed arrows show that coefficients did not differ significantly from 0 (*p* > 0.05). The model is assessed using the Goodness of Fit (GoF) statistic. Fluvo-aquic soil (**A**) and black soil (**B**).

We conducted ABT analysis to determine the specific contribution of soil physicochemical and soil biological properties to bacterial diversity and community structure. Key soil variables affecting bacterial diversity and community structure varied in the two soils. In fluvo-aquic soil, AP was the most influential factor in shaping bacterial diversity (31%) and community structure (46%) (Figure 6A,B; Supplementary Table S1), whereas in black soil, soil pH played a substantial role in shaping bacterial diversity (55%) and community structure (35%) (Figure 6C,D). In addition to ABT analysis, the relationship among soil variables, soil bacteria and maize yield was determined using the PLSPM. The results of the PLSPM revealed that, in fluvo-aquic soil, N contents, including TN and NO_3_^−^ N, had significant positive effects on maize yield (0.63) (Figure 7A). Microbial biomass including MBN and MBC (0.41) had significant positive relationships with crop yield. The effect of soil pH on crop yield had indirect negative relationship, but not significant (Supplementary Table S2). AP had significant positive and negative effects on bacterial community structure (0.54) and diversity (−0.58), respectively. In black soil, N contents had significant positive relationship with maize yield (0.93) (Figure 7B). Soil pH had a significant positive effect on bacterial diversity (0.65) and community structure (0.52). Microbial biomass, including MBC and MBN, had a significant positive effect on bacterial community structure (0.43) and diversity (0.34), respectively.

## 4. Discussion

### 4.1. Soil Chemical Properties and Biochemical Properties in Relation to Maize Yield 

The recommended and conventional fertilization significantly altered the physiochemical properties of fluvo-aquic and black soils (Table 2). Earlier studies have shown that anthropogenic N enrichment increases soil TN and SOC [31]. However, elevated N fertilization can negatively influence synthesis and breakdown of soil organic matter (SOM) due to the essential coupling of C and N cycling [1]. A decrease in soil pH in response to N addition was observed particularly in CF treatments, and earlier studies demonstrated that N addition causes soil acidification due to the substantial uptake of base cations while at the same time, crops also release equivalent H^+^ to the soil. Additionally, H^+^ is liberated during the oxidation of NH_4_^+^ N to NO_3_^−^ N [32]. Soil acidification due to N enrichment also depends on the N addition rate, i.e., the decline in soil pH is more pronounced when the N rate is higher [33]. Different soils have distinct initial characteristics and edaphic properties that may induce varied effects in response to N addition [34]. For example, we found that in fluvo-aquic soil, a mild decrease in soil pH due to N addition (Table 2) could be ascribed to calcareous soil that has a greater buffering capability in terms of soil pH reduction [35]. The AP in our study was significantly reduced as a result of N addition. Earlier studies showed that soil acidification as a result of N fertilization could make inorganic P unavailable due to the adsorption to the surfaces of minerals [36].

The enhanced soil nutrient bioavailability due to inorganic fertilization, especially N fertilizer, is associated with crop yield [37]. In particular, PLSPM showed that soil biochemical properties attributes were mediated by N addition that can directly or indirectly impact maize grain yield by mediating soil bacterial communities with consequences for crop production. For instance, NO_3_^−^ N, TN, MBC, and MBN levels were the key drivers regulating maize grain yield either interactively or individually, through mediating soil bacteria (Figure 7). Soil MBN has a critical role in soil fertility which can influence availability of N for plant uptake and immobilization of mineral N [38], because MB pools can provide a conducive environment and energy for the release and production of soil extracellular enzymes [39]. Other studies suggest that recommended levels of microbial biomass C and N pools and community structure have a significant role in maintaining the productivity and sustainability of terrestrial ecosystems by mediating various soil biogeochemical processes [9,40]. Furthermore, it has been well been well established that the highest productivity may induce the observed effects on microbes, supplying more labile C through crop residues and root exudates [41,42], where it may enhance soil organic matter decomposition by free-living soil saprotrophs and consequently increase in nutrient availability [43].

### 4.2. Bacterial Alpha Diversity

We used the Shannon index [29] and Chao1 [30] to analyze microbial diversity and richness, respectively, because they have been highly recommended for determining microbial alpha diversity [44]. Microbial diversity is an important soil indicator, greater biodiversity more stable will be the system and improve microbial activities and processes [9]. It is worth mentioning that across both sites bacterial diversity and richness was not significantly reduced in plots with recommended N application (Figure 2). The subsidy-stress hypothesis states that higher levels of N addition results in reduced diversity, while moderate levels of amendment can promote microbial diversity [11,12]. By contrast, N addition in CF treatments substantially reduced bacterial diversity and richness. It is well documented that elevated amounts of N input results in reduction of bacterial diversity [10]. In addition, a study showed that the conventional farming system has elevated resource inputs, higher losses, and reduced rate of soil internal regulatory processes, which eventually deplete soil life [40] reported that the conventional farming system has elevated resource inputs, higher losses, and reduced rate of soil internal regulatory processes, which eventually deplete soil life. 

Soil microorganism have substantial impacts on soil ecosystems and govern numerous important soil processes, for instance, many processes in the N cycle are exclusively carried out by soil microorganisms (e.g., Biological N fixation, nitrification, and denitrification). These processes are extremely important for proper ecosystem functioning because N availability has a profound role in plant productivity [45]. Furthermore, soil biological processes determine the potential of soils to sequester C, it is generally accepted that the soil with higher proportion of bacteria and fungi, the potential for C sequestration increases [46]. Therefore, any alteration or reduction in diversity of functional microbial groups as a result of excessive N fertilization can hamper soil ecosystem functions. The reduction in microbial diversity in response to N fertilization also depends on soil pH; for example, the impact of N addition on microbial diversity was more pronounced when N was added to alkaline soils than to acidic soils [19]. A previous study further confirmed that each type of soil has its own properties, such as indigenous microbial activity, and diverse edaphic properties, which may results in unquantifiable and different impacts on N addition on soil responses [34]. For instance, different types of soil have distinct water conditions, texture, pore space, and aeration, which can impact the performance of N addition on soil micro-biota [47].

### 4.3. Bacterial Community Structure

Shifts in taxonomic composition showed that, across the two soils relative abundance of copiotrophic groups such as phyla Proteobacteria and Firmicutes increased while, oligotrophic groups such as Acidobacteria, Nitrospirae, and Chloroflexi decreased in response to N addition in RF and CF treatments (Figure 4), and these results align well with previous studies [16,19]. The shifts in bacterial composition following N addition can be explained by the copiotrophic hypothesis [6], in which copiotrophic groups survive best under nutrient-rich conditions, while oligotrophic groups thrive well under low-C conditions. We found a positive relationship between soil pH and abundance of acidobacteria (Supplementary Figure S1). Further deeper analysis at order level also revealed that acidobacteria *subgroup_6* and *Acidobacteriales* were affiliated to plots with higher soil pH (Figure 5B; Table 2). These results were consistent with those of [48], that acidobacteria subgroup 6 had a positive correlation with soil pH. In addition, Wang et al. [17] findings also revealed that Acidobacteria had positive relationship with soil pH. Alternatively, another researcher demonstrated that abundance of acidobacterial groups (e.g., 7, 17) had negative relationship with soil pH [49]. The differences in these results are likely due to different soil types because, different soil types have their own unique traits, such as indigenous microbial properties and distinct edaphic traits, which can induce distinct effect of N fertilization on soil responses [34]. Furthermore, other researchers added that the responses of bacteria at lower taxonomic levels within the same phylum can be different [50].

The order levels taxonomic analyses showed that bacterial orders Bacillales and Rhodospirillales were significantly enhanced in RF N182 treatment (Figure 5A). Earlier studies showed that Bacillales were tightly related to N fertilization [16], which aligns well with our results. Members belonging to Bacillales have numerous ecological roles, such as improving plant growth [51], fixing N [52], and acting as biological control against *Ralstonia solanacearum*, which causes bacterial wilt of plants [53]. Moreover, some groups belonging to Rhodospirillales have substantial role in fixing molecular N [52], and enhanced abundance of such bacterial groups can be beneficial in the N cycling. The dramatic increase in Nitrosomonadales in CF N225 treatment (Figure 5A), can be ascribed to the fact that this treatment received a higher N fertilization rate and it is generally believed that high N addition can enhance the abundance of nitrifying bacteria [54]. We found that Xanthomonadales were significantly altered in both the CF N225 and CF N251 treatments. Studies have demonstrated that some species belonging to the order Xanthomonadales are capable of denitrification [55], and that a higher abundance of such species pose negative impacts on ecosystem stability. Elevated N inputs (CF N225 and CF N251 treatments) have significantly reduced order Rhizobiales, indicating higher N input severely impact symbiotic N-fixing bacteria. One of the main reasons in the reduction of these microbial groups can be that; high amount of external N input was able to inhibit N_2_ fixation and, ultimately, the presence of these organisms [56]. The dramatic reduction in aforementioned functional microbes affiliated to CF treatments receiving N suggest that this fertilization approach can possibly have adverse impact on ecosystem functions. 

### 4.4. Relationship between Soil Properties versus Bacterial Diversity and Community Structure

The interaction between soil chemical properties, biological properties, and microbial communities has resulted in a debate about the mechanisms in nutrient cycling and ecosystem processes, and it is very imperative to understand these complex interactions for proper biome functioning. Numerous soil properties play critical roles in shaping bacterial community structure and diversity when the soil is subjected to fertilization. For instance, changes in TN, SOC, and AP are associated with shifts in bacterial communities [5,7,37]. In the present study, the ABT model indicated that AP was the most instrumental important soil variable affecting both bacterial diversity and community structure in fluvo-aquic soils (Figure 6A,B). Alterations in bacterial community structure and diversity due to soil P availability have been previously reported. For example, Ling et al. [16] showed that bacterial diversity was not affected by P availability, but that richness and community structure were tightly related to soil AP. PLSPM results of this study further showed that AP negatively impacted soil microbial diversity (Figure 7A). A study demonstrated that soil microbial diversity declined in response to P input in arable soils of northern China [57]. Notably, both P and N inputs caused soil acidification [16], and it is widely accepted that soil acidification results in the leaching of Mg^2+^, Ca^2+^, and Na^+^ and mobilization of Al^3+^ [8]. Therefore, microbial growth is restricted due to the unavailability of Mg^2+^, Ca^2+^ and Na^+^ and the enhanced availability of Al^3+^ [8], and these conditions ultimately impact microbial diversity. Another study noted that P induced alterations in the microbial community through osmotic effects rather than pH effects [58]. Furthermore, it was demonstrated that P input could reduce total soil C and light fraction soil C and consequently regulate the bacterial community [59] demonstrated that P input could reduce total soil C and light fraction soil C and consequently regulate the bacterial community, and it is well documented that alterations in soil microbial community structure are tightly related to variations in soil C distribution and storage [7]. 

In this study, we further found that soil pH was positively linked with both bacterial community structure and diversity in black soils (acidic soil) (Figure 6C,D and Figure 7B). Previous studies have shown that soil pH exerts a great impact on shaping bacterial community structure and diversity [10,48]. Notably, Zeng et al. [15] showed that soil acidification rather than N availability was responsible for alteration in bacterial community structures, whereas, N availability was responsible for variation in bacterial richness. Conversely, it was claimed that N availability rather than soil pH induced changes in soil microbial communities [60]. It is well established that N addition causes soil acidification due to the large uptake of base cations, while at the same time, crops also release equivalent H^+^ to the soil. Additionally, H^+^ is liberated during the oxidation of NH_4_^+^ N to NO_3_^−^ N, and as a result of these processes, biodiversity is seriously diminished [4]. Bacterial diversity was inconsistent across different ecosystems, and soil pH was the critical factor responsible for explaining the majority of variation [48]. Taken together, these findings suggest that important soil variables linked with bacterial diversity and community composition varied in different soil types, implying that soil edaphic factors play profound role in determining microbial communities.

## 5. Conclusions

To safeguard the planet earth from adverse effects of over-fertilization at the same time as producing a higher yield to feed the mouths of the ever-increasing population, it is recommended fertilization could be a potential alternative farming system. Our results demonstrated that conventional fertilization, which is characterized by elevated N input, not only deteriorated soil quality by soil acidification, but also depressed bacterial diversity. It is well recognized that bacterial diversity is imperative for ecosystem functions and stability. Therefore, if N enrichment consistently increased in the future, then the reduction in bacterial diversity would worsen, which will eventually hamper ecosystem functions. Conversely, recommended fertilization successfully maintained bacterial diversity. Recommended fertilization also improved beneficial bacterial taxa which perform a remarkable role in ecosystem functions. Meanwhile, our results revealed that AP and pH are determining soil factors that couple with bacterial diversity and bacterial community structure in fluvo-aquic and black soil, respectively. Our study provides novel information pertaining to how belowground microbial communities respond to recommended and conventional fertilization regimes. Furthermore, in comparison with community-level changes in soil bacteria, the important high-magnitude biomarkers differentiating bacterial communities under recommended and conventional fertilization regimes require further detailed examination in future studies for sustainable crop production.

## Figures and Tables

**Table 1:** Detailed illustration of fertilizer application for maize crop.

Fertilization	N-P-K	N-P-K	N-P-K	N-P-K
Fluvo-Aquic Soil	RF N0	RF N182	CF N0	CF N225
	0–73–70	182–73–70	0–120–50	225–120–50
Black Soil	RF N0	RF N180	CF N0	CF N251
	0–75–90	200–75–90	0–145–100	251–145–100

RF = Recommended fertilization; CF = Conventional fertilization; N-P-K = nitrogen, phosphorus, and potassium fertilizers (kg ha^−1^) respectively.

**Table 2:** Impacts of recommended and conventional fertilization regimes on soil chemical and biological properties.

Treatments	TN	SOC	AP	NO_3_^−^	NH_4_^+^	MBN	MBC	pH	C:N
Fluvo-Aquic Soil									
RF N0	1.18 b	20.08 a	16.61 b	6.03 c	1.38 a	271 a	74.17 b	8.05 ab	16.90 a
RF N182	1.28 a	21.19 a	14.87 b	26.50 b	1.48 a	329 a	88.81 a	8.00 bc	16.46 a
CF N0	1.22 ab	20.60 a	24.40 a	11.44 c	1.37 a	273 a	77.64 ab	8.11 a	16.80 a
CF N225	1.27 ab	21.07 a	18.80 b	34.39 a	1.50 a	299 a	79.15 ab	7.97 c	16.55 a
Significance	*	ns	*	***	ns	ns	ns	**	ns
Black Soil									
RF N0	1.00 b	22.76 a	53.81 b	2.51 c	1.28 bc	62 a	13.30 a	5.82 a	22.55 a
RF N180	1.21 a	23.99 a	43.02 c	13.44 a	3.04 a	35 b	10.43 ab	5.56 ab	19.80 b
CF N0	1.01 b	23.79 a	62.43 a	2.38 c	1.10 c	77 a	9.98 b	5.91 a	23.54 a
CF N251	1.22 a	24.18 a	52.80 b	9.19 b	2.49 ab	21 b	5.15 c	5.23 b	19.73 b
Significance	***	ns	**	***	**	**	*	**	***

Data are the means, n = 4. Different letters indicate significant differences among treatments. TN = Total Nitrogen (g kg^−1^); SOC = Soil Organic Carbon (g kg^−1^); AP = Available Phosphorus (mg kg^−1^); NO_3_^−^ = Nitrate N (mg kg^−1^); NH_4_^+^ = Ammonium N (mg kg^−1^); MBN = Microbial Biomass Nitrogen (mg kg^−1^); MBC = Microbial Biomass Carbon (mg kg^−1^); C:N = Carbon; Nitrogen.* *p* ≤ 0.05; ** *p* ≤ 0.01; *** *p* ≤ 0.001; ns = not significant

**Table 3:** Effect of fertilization, soil type, and their interaction on soil biochemical traits, maize yield, and bacterial alpha diversity indices.

Treatments	TN	SOC	AP	NO_3_^−^	NH_4_^+^	MBN	MBC	pH	C:N	Yield	Chao1 Index	Shannon Index
Fertilization	**	ns	**	**	**	ns	ns	**	**	**	**	**
Soil Type	**	**	**	**	**	**	**	**	**	**	**	**
Interaction	**	ns	ns	**	**	*	**	**	**	**	**	**

* *p* ≤ 0.05; ** *p* ≤ 0.01; ns = not significant.

## Supplementary Materials

The following are available online at https://www.mdpi.com/2076-2607/8/8/1193/s1, Figure S1: Pearson correlation analysis between soil properties and most abundant bacterial phyla in fluvo-aquic soil (A) and blacks soil (B). Red and blue circles represent negative and positive correlation, respectively. Acid. = Acidobacteria; Act. = Actinobacteria; Bac. = Bacteroidetes; Cyan. = Cyanobacteria; Chlo. = Chloroflexi; Firm. = Firmicutes; Gemm. = Gemmatimonadetes; Nitr. = Nitrospirae; Prot. = Proteobacteria, Table S1: Contributions of the first five most important driving forces to the variation in the abundance, diversity and community structure of soil bacteria, Table S2: The direct and indirect relationships between variables. The path coefficients are calculated by PLS-PM after 1000 bootstrap.
