# Supplementary material for: How Do Soil Bacterial Diversity and Community Composition Respond under Recommended and Conventional Nitrogen Fertilization Regimes?
Source: Microorganisms. 2020 Aug 5;8(8):1193. doi: 10.3390/microorganisms8081193 (PMC7466009; doi:10.3390/microorganisms8081193)
Supplement: Supplementary file 1 [file microorganisms-08-01193-s001.zip › microorganisms-868024-supplementary/supplementry information.docx]

Supplementary Material

How do soil bacterial diversity and community composition respond under recommended and conventional Nitrogen fertilization regimes?

**Sami Ullah ^1^, Ping He^1,*^, Chao Ai^1^, Shicheng Zhao^1^, Wencheng Ding^1^, Dali Song^1^, Jiajia Zhang^1^, Shaohui Huang^1^, Tanveer Abbas^1^, and Wei Zhou^1^**

^1^ Ministry of Agriculture Key Laboratory of Plant Nutrition and Fertilizer, Institute of Agricultural Resources and Regional Planning, Chinese Academy of Agricultural Sciences (CAAS), Beijing 100081, China

*** Correspondence:**Corresponding Author
heping02@caas.cn

**Table S1**

Contributions of the first five most important driving forces to the variation in the abundance, diversity and community structure of soil bacteria.

|  | Total explained variation (%)^a^ | Variation explained by (%)^b^ | | |  |  |
| --- | --- | --- | --- | --- | --- | --- |
| Fluvo-aquic soil | |  |  |  |  |  |
| Diversity | 85.73 | AP (31.8)** | NO_3_-N (27.26)** | NH_4_-N (12.87)** | SOC (7.21)** | TN (6.59)** |
| Community structure | 85.88 | AP (46.79)** | NH_4_-N (14.16)** | NO_3_-N (11.97)** | SOC (7.16)** | MBC (5.8)** |
|  |  |  |  |  |  |  |
| Black soil |  |  |  |  |  |  |
| Diversity | 91.40 | pH (54.99)** | MBN (15.99)** | TN (12.24)** | SOC (4.27)** | MBC (3.91)** |
| Community structure | 93.04 | pH (34.87)** | TN (29.68)** | MBN (17.94)** | SOC (6.92)** | AP (3.63)** |

^a^ Total explained variance was determined by aggregated boosted tree (ABT) analysis including all the retained explanatory variables.

^b^ The respective contributions were calculated by taking into account all retained explanatory variables.

Abbreviations: AP., available phosphorus; NO_3_-N., nitrate nitrogen; NH_4_-N., ammonium nitrogen; SOC., soil organic carbon; TN., total nitrogen MBN., microbial biomass nitrogen; MBC., microbial biomass carbon

Significance levels (*P*) : ***P* < 0.01

**Table S2**

The direct and indirect relationships between variables. The path coefficients are calculated by PLS-PM after 1000 bootstrap.

| Fluvo-aquic soil | |  |  |  | Black soil |  |  |  |
| --- | --- | --- | --- | --- | --- | --- | --- | --- |
| Relationships | Direct | Indirect | Total |  | Relationships | Direct | Indirect | Total |
| AP -> N | 0.000 | 0.000 | 0.000 |  | AP -> N | 0.000 | 0.000 | 0.000 |
| AP -> PH | 0.000 | 0.000 | 0.000 |  | AP -> PH | 0.000 | 0.000 | 0.000 |
| AP -> MB | 0.000 | 0.000 | 0.000 |  | AP -> MB | 0.000 | 0.000 | 0.000 |
| AP -> DIV | -0.582 | 0.000 | -0.582 |  | AP -> DIV | -0.088 | 0.000 | -0.088 |
| AP -> BAC | 0.542 | 0.000 | 0.542 |  | AP -> BAC | 0.056 | 0.000 | 0.056 |
| AP -> YLD | 0.000 | -0.132 | -0.132 |  | AP -> YLD | 0.000 | -0.017 | -0.017 |
| N -> PH | 0.000 | 0.000 | 0.000 |  | N -> PH | 0.000 | 0.000 | 0.000 |
| N -> MB | 0.000 | 0.000 | 0.000 |  | N -> MB | 0.000 | 0.000 | 0.000 |
| N -> DIV | -0.391 | 0.000 | -0.391 |  | N -> DIV | 0.096 | 0.000 | 0.096 |
| N -> BAC | 0.341 | 0.000 | 0.341 |  | N -> BAC | -0.053 | 0.000 | -0.053 |
| N -> YLD | 0.625 | -0.082 | 0.543 |  | N -> YLD | 0.934 | 0.018 | 0.951 |
| PH -> MB | 0.000 | 0.000 | 0.000 |  | PH -> MB | 0.000 | 0.000 | 0.000 |
| PH -> DIV | 0.161 | 0.000 | 0.161 |  | PH -> DIV | 0.646 | 0.000 | 0.646 |
| PH -> BAC | 0.310 | 0.000 | 0.310 |  | PH -> BAC | 0.524 | 0.000 | 0.524 |
| PH -> YLD | 0.000 | -0.097 | -0.097 |  | PH -> YLD | 0.000 | 0.004 | 0.004 |
| MB -> DIV | 0.015 | 0.000 | 0.015 |  | MB -> DIV | 0.343 | 0.000 | 0.343 |
| MB -> BAC | -0.131 | 0.000 | -0.131 |  | MB -> BAC | 0.432 | 0.000 | 0.432 |
| MB -> YLD | 0.407 | 0.037 | 0.444 |  | MB -> YLD | -0.043 | -0.018 | -0.061 |
| DIV -> BAC | 0.000 | 0.000 | 0.000 |  | DIV -> BAC | 0.000 | 0.000 | 0.000 |

**Figure S1.** Pearson correlation analysis between soil properties and most abundant bacterial phyla in fluvo-aquic soil (A) and blacks soil (B). Red and blue circles represent negative and positive correlation, respectively. Acid. = Acidobacteria; Act. = Actinobacteria; Bac. = Bacteroidetes; Cyan. = Cyanobacteria; Chlo. = Chloroflexi; Firm. = Firmicutes; Gemm. = Gemmatimonadetes; Nitr. = Nitrospirae; Prot. = Proteobacteria.
